# Collaborative engagement of Hispanic communities in the planning, conducting, and dissemination of assistive technology research

**DOI:** 10.1017/cts.2020.534

**Published:** 2020-09-04

**Authors:** Elsa M. Orellano-Colón, Marta Rivero-Méndez, Claudia X. Boneu-Meléndez, Solymar Solís-Báez, Arelí León-Astor, Mariolga Juliá-Pacheco, María del Mar Santiago-Cruz, Jeffrey W. Jutai

**Affiliations:** 1 Occupational Therapy Program, University of Puerto Rico Medical Sciences Campus, San Juan, Puerto Rico; 2 School of Nursing, University of Puerto Rico Medical Sciences Campus, San Juan, Puerto Rico; 3 Office of Environmental Quality, Health, and Occupational Safety, University of Puerto Rico Medical Sciences Campus, San Juan, Puerto Rico; 4 Corporación del Proyecto ENLACE del Caño Martín Peña, San Juan, Puerto Rico; 5 Caño Martín Peña Community Land Trust, San Juan, Puerto Rico; 6 University of Ottawa and LIFE Research Institute, Ottawa, Canada

**Keywords:** Assistive technology, community engagement, community stakeholders, Hispanic, Puerto Rico, translational research

## Abstract

**Introduction:**

Community engagement (CE) is critical for research on the adoption and use of assistive technology (AT) in many populations living in resource-limited environments. Few studies have described the process that was used for engaging communities in AT research, particularly within low-income communities of older Hispanic with disabilities where limited access, culture, and mistrust must be navigated. We aimed to identify effective practices to enhance CE of low-income Hispanic communities in AT research.

**Methods:**

The community stakeholders included community-based organizations, the community healthcare clinic, the local AT project, and residents of the Caño Martín Peña Community in San Juan, Puerto Rico. The CE procedures and activities during the *Planning the Study Phase* comprised working group meetings with stakeholders to cocreate the funding proposal for the study and address the reviewers’ critiques. During the *Conducting the Study Phase*, we convened a Community Advisory Board to assist in the implementation of the study. During the *Disseminating the Study Results Phase*, we developed and implemented plans to disseminate the research results.

**Results:**

We identified seven distinct practices to enhance CE in AT research with Hispanic communities: (1) *early and continuous input*; (2) *building trusting and warm relationships through personal connections*; (3) *establishing and maintaining presence in the community*; (4) *power sharing*; (5) *shared language*; (6) *ongoing mentorship and support to community members*; and (7) *adapting to the changing needs of the community*.

**Conclusion:**

Greater attention to CE practices may improve the effectiveness and sustainability of AT research with low-income communities.

## Introduction

Functional disabilities (defined as difficulties with performing daily life activities) that result from chronic conditions increase older adults’ vulnerability to diminished quality of life, loss of independence, higher healthcare costs and service utilization, and negative health outcomes [1–3]. Unfortunately, prevalence of disabilities differed across race and ethnicity. This is, older adults of racial/ethnic minority background are generally at higher risk than non-Hispanic Whites to report functional disabilities [4,5]. Specifically, there is a striking disparity in disability rates among older Puerto Ricans, with older Hispanics living in Puerto Rico reporting a substantially higher rates of functional disabilities (28.9%) compared to 14.2% of older adults from the continental US [6].

Assistive technology (AT) devices can help reduce these disparities [7]. Randomized controlled trials [8–10] provide evidence that relatively inexpensive AT devices such as jar openers, sock aids, and elevated toilet seats can slow functional decline with aging, improve well-being, and ability to stay in their homes as long as possible. Moreover, national data reveal that successful accommodations through AT among those who experience declines in capacity may be an effective means of promoting participation and well-being in later life [11]. However, Hispanics are among the least likely to use and access AT devices [12–14]. Given the aging of the population, if issues of non-adoption and lack of access to AT are left unaddressed, older Hispanics’ loss of functional independence and healthcare costs will continue to escalate.

To address health inequities, the National Institutes of Health, the Patient Centered Outcome Research Institute, and the Robert Wood Johnson Foundation increasingly promote engagement of the communities whose health will be directly affected in research that they fund. Community engagement (CE) is an approach that encourages meaningful community input into the design, implementation, and dissemination of research to improve research quality and outcomes [15]. The CE approach improves research by identifying relevant questions, enlisting community resources, and generating knowledge that is more easily transferable and usable by the affected community [16]. As CE is becoming more widely expected as a feature of ethical collaborative research, it is important to identify good CE practices and be able to provide specific descriptions of how they contribute to the effectiveness of CE. In the AT research field, there is some evidence that community involvement can help create a better match between what users want and need and the available AT, which in turn could promote adoption and diffusion of AT. However, there has been little guidance about the best ways to include communities in AT research. Moreover, there are many challenges in translating CE principles into community-based AT research in low-income Hispanic communities. These challenges include (1) lack of AT researchers’ understanding and experience with engaging communities; (2) skepticism about research in these communities; (3) power differences in which the research process is still mainly controlled by the researchers; and (4) a limited group of stakeholders being involved in rehabilitation research [17,18].

Mistrust of academic institutions and researchers is common among minority populations. Historically, many minority’s communities have been mistreated or taken advantage of, by being used like “guinea pigs” without receiving the health benefits that results from research [19]. Other times, researchers generate unrealistic solutions to community health problems as a result of not engaging community members in the research process. This calls for a process of insertion and construction of relationships with communities from a framework of human rights [20]. As stated by Mariolga Juliá-Pacheco, the director of the Office of Social Development and Citizen Participation of the *Corporación del Proyecto Enlace* of the Caño Martín Peña (CMP): *“communities must be recognized as subjects with rights that need to be validated in all research with human subjects”* (personal communication, May 12, 2020). Given these communities rights, it is imperative to engage community members as much as possible in the design, implementation, and dissemination of research studies. For this to happen, there should be a space for researchers to get to know and understand the communities and its members, their practices, and their reality at first hand. This requires a high degree of empathy as well as an exchange of knowledge that equally distributes the power among the researchers and community members. A distribution of power based on a deep and empathetic knowledge of the community will build a solid foundation to tailor research studies based on communities’ real health needs that will result in relevant and effective communities’ health practices.

In spite of the importance of CE in health research, the AT field is lacking richly descriptive information of practical and real-world examples of how to apply the principles of CE. This article contributes to the AT evidence base by describing an approach to CE derived from an NIH-funded study of disparities in the adoption and use of AT by older Hispanics living in Puerto Rico. We aimed to identify effective practice guidance to enhance CE of low-income Hispanic communities in AT research.

## Materials and Methods

### Design

The CE practices emerged from the first year of an ongoing 2 years sequential mixed-methods study which started on August 1, 2019. The aims of this study are to identify the functional disabilities of Hispanic older adults living in a low-income community in Puerto Rico as well as to explore the gender differences in factors influencing the use of AT. The first quantitative phase of this study consisted of the collection of cross-sectional data from a sociodemographic questionnaire and a functional disability measure (PROMIS® Physical Function Short Form-20) among 211 randomly selected older Hispanics living in a low-income community in PR. The second qualitative phase is currently being conducted and consists of in-depth, semi-structured interviews assisted by videos showing older people using AT to encourage a more productive process, with a purposive sample of 12 men and 12 women with the highest levels of functional disabilities reported in the quantitative phase.

The community that participated in this study is the CMP in San Juan, Puerto Rico. The CMP comprises eight communities grouped by the PR Law 489-2004, to guarantee citizen participation processes that result in an enhanced quality of life of more than the 20,000 residents of this community. The CMP has all of the essential features that define community in relation to research, including common culture and shared knowledge, health-related common culture, mechanism for healthcare priority setting, geographic localization, and self-identification as a community [21].

### Procedures

**Fig. 1.**
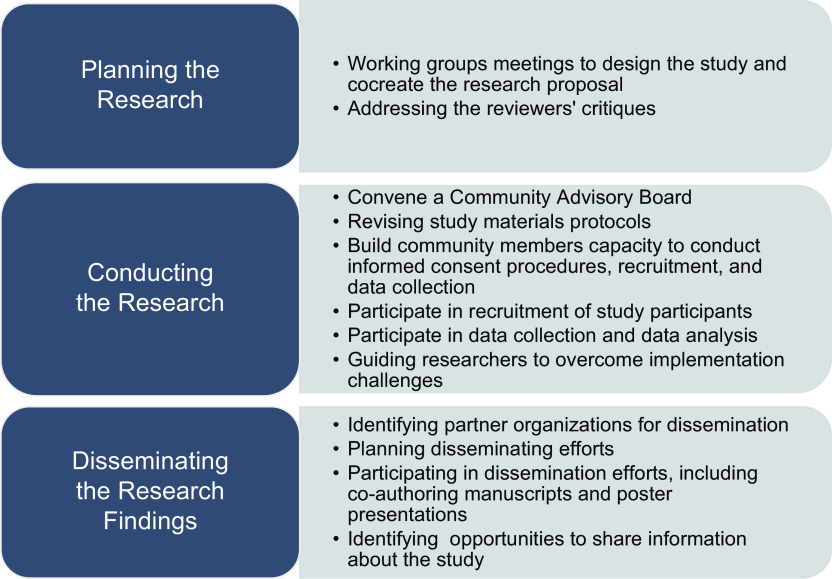
Community engagement activities that took place within each phase of the research process of the study.

We employed the CE procedures and activities throughout the following research phases: (1) *Planning the Study*; (2) *Conducting the Study*; and (3) *Disseminating the Study Results.* Fig. 1 depicts the complete list of activities conducted within each phase of the research process of this study. During *the Planning the Study Phase*, we conducted four working group meetings with stakeholders to cocreate the funding proposal for the study in its early stages to assure the relevance of the research question and study procedures. The stakeholders who participated in this phase were the following: two women from the *G-8, Inc.* (group of leaders of the eight communities of the CMP); the women director of the Office of Social Development and Citizen Participation of the *Corporación del Proyecto Enlace* of the CMP (a governmental corporation with the mission to oversee and implement the CMP District Plan); the women coordinator of the G-8, Inc. from the *Fideicomiso de la Tierra Community Land Trust*; and two older women and one older men with functional disabilities and residents of the CMP. Working group meetings time lengths were approximately 2 hours. Two meetings were conducted in the conference room of the *Corporación del Proyecto Enlace* of the CMP and two were conducted in the G-8, Inc. headquarters. During this phase, we also obtained and incorporated the stakeholders’ input to address reviewers’ critiques in preparing a resubmission of the application. While conducting the working groups, the researchers served as facilitators using open-ended questions to stimulate an active and open discussion of topics. In addition, each meeting had mutually agreed established goals, and action plans were developed together.

During the *Conducting the Study Phase* (once the study was approved by the Institutional Review Board of the University of Puerto Rico Medical Sciences Campus), we convened a Community Advisory Board (CAB) to revise recruitment and data collection materials, provide guidance on how to overcome implementation challenges, and participate in data analysis. The CAB was composed by the stakeholders who participated during the *Planning the Study Phase*, with the addition of one male Program Official and one female Community Organizer from the *Corporación del Proyecto Enlace*; two female representatives from the HealthproMed community health clinic; one female occupational therapy representative from the Puerto Rico Assistive Technology Program; and five female community interviewers. All three CAB’s meetings during year one were conducted in the conference room of the *Corporación del Proyecto Enlace.* CAB’s members did not receive monetary incentives for their participation in the meetings; however, a lunch was served in each meeting. In this phase, we also built the community interviewers’ capacity to conduct the study’s informed consent procedures, recruitment and data collection through a 4 days, 8 h training on research ethics for community members, the study’s goals, significance, and relevance, and skilled training on participants’ recruitment and data collection methods. The interviewers’ competence on administrating the informed consent procedures, participants’ recruitment, and data collection process were assessed by the researchers of this study and using the Interviewer Competence Scale which was developed for the purpose of this study. This scale used a three points system scale (2 = Meets the expectations; 1 = Needs improvement; 0 = Inadequate performance) to assess their levels of competence in areas such as organization (have all the required materials for conducting the interviews and use the interview materials in the correct sequence); communication skills (use simple and pertinent answers to participants concerns or questions); and assessment skills (correctly provides the instruments’ instructions and scores and interpret the instruments results). The community interviewers received a compensation of $240 for their participation in the 4 days training sessions, $25.00 for each completed participant’s interview, and $16.00 for their participation in each one of the three meetings with the researchers with a duration of 2 hours each. All community interviewers provided their written authorization to use photographs and videos of them taken as part of this study in publications and presentations.

During the *Disseminating the Study Results Phase*, the CAB members guided the researchers to identify partner organizations for dissemination, develop and implement plans to disseminate the research results, and identifying opportunities. During this phase, an important CE activity was also stakeholders’ participation in dissemination efforts, such as co-authoring manuscripts and poster presentations.

### Data collection and analysis

Data from the principles of CE with the community members and stakeholders were collected by the occupational therapy and nursing researchers, as well as by the research assistant of this study. These researchers performed qualitative observations and captured information through field notes during each CE activity that was conducted within each research phase. Field notes recorded the researchers’ and community members’ interactions and possible principles based on these interactions. Data were analyzed by these researchers during their weekly staff meetings by conducting a reflexive thematic analysis [22] of the observations and field notes. Coding was undertaken by the researchers using a collaborative process in which codes were developed, collapsed, and promoted into themes from clustering together similar codes. The themes that emerged from this analysis captured a shared meaning organized around the principles of CE for engaging the CMP stakeholders in the research process of this study.

## Results

Sixteen community stakeholders have participated in the development and implementation of the AT study which is still being conducted until July 31, 2021. Fourteen (86%) of these participants are women. The community–academic partnership has resulted in the successful recruitment of 213 participants, submission of two abstracts with community stakeholders as co-authors, and one presentation in a national symposium. The implementation of this research CE procedures and activities resulted in the following important practices to engage Hispanic community stakeholders in our AT study: (1) *early and continuous input*; (2) *building trusting and warm relationships through personal connections*; (3) *establishing and maintaining presence in the community*; (4) *power sharing*; (5) *shared language*; (6) *ongoing mentorship and support to community members*; and (7) *adapting to the changing needs of the community*. These practices were identified through observation of the process of CE with the community members and stakeholders of the CMP communities.

### Early and Continuous Input

It was crucial to engage community stakeholders’ input early in the research process. Early community input was obtained during our initial meetings with the CMP stakeholders, but after investing a considerable amount of these meetings time to deeply learn about the CMP communities’ history, socioeconomic issues, community development activities, and achievements. These meetings were conducted three times a year, starting on 2016, with the director of the *Proyecto Enlace* Office of Citizen Participation and Social Development, representatives from the *Fideicomiso de la Tierra Community Land Trust*, and the G-8 Inc. organization which groups the community leaders from of the eight communities comprising the CMP. After understanding the CMP history and needs, the researchers were able to contextualize the community input in order to successfully incorporate the community voices throughout the research process.

Early community input enhanced the relevance and success of our study in several ways. This is, during the *Planning the Research Phase*, the stakeholders validated the significance of the study aims for addressing the disability disparities of the older Hispanics living in this community. They participated in the recruitment and data analysis of the implementation of the preliminary pilot study and provided valuable input that strengthened the significance and recruitment methods for addressing the reviewers’ critiques in the resubmission process. During the *Conducting the Research Phase*, continuous community input was obtained from the CAB that was convened in 2019 to guide the researchers during the implementation of this study. The CAB input helped alleviate participants’ recruitment and implementation challenges, resulting in several modifications to the research protocol. For example, we changed the participants’ incentives method from gift cards to cash to accommodate the payment method used by older adults from this community. Based on the CAB recommendations, we engaged the G-8 Inc. leaders in the process of recruiting the community interviewers. Moreover, the CAB recommendations of using the G-8 identification vest during participants’ recruitment and keeping the community leaders informed about the recruitment schedule, assured interviewers and researchers’ safe access to the community during recruitment and data collection. Input from the community interviewers was also instrumental during the *Conducting the Research Phase*. For instance, the community interviewers input enhanced the quality of the collected data by adding important items to the sociodemographic questionnaire that were unknown by the researchers, such as the Temporary Assistance for Needy Families (TANF) Program as one of the sources of income received by older adults living in the CMP communities. Finally, during the *Disseminating the Results of the Research Phase*, the CAB provided valuable input to disseminate the findings of this study in the community’s newspaper and in the community health clinic’s television through the use of infographics to visually present the results in a more accessible format.

### Building Trusting and Warm Relationships Through Personal Connections

**Fig. 2.**
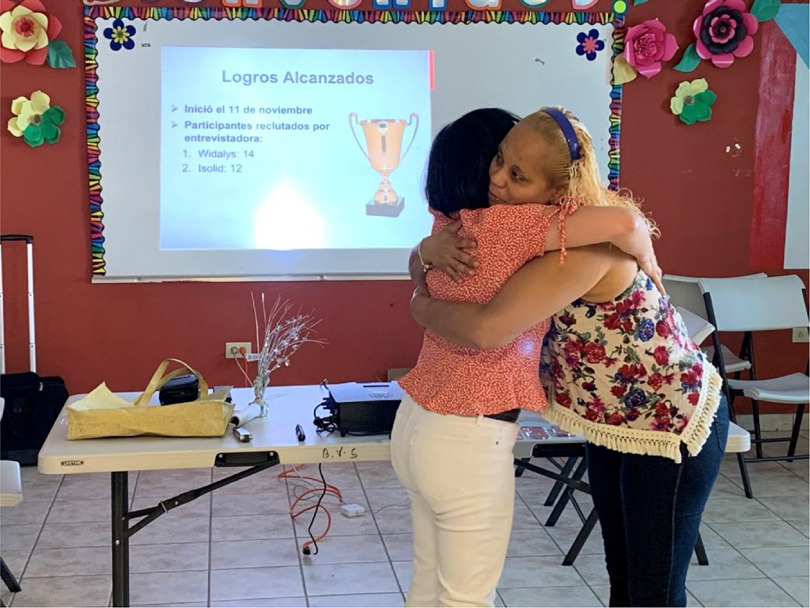
Close physical contact between the researchers and community members in celebration of the achievements in the recruitment process.

Mutual trust between the researchers and the community was built overtime and nurtured through several means. The early honest and transparent consultations with community leaders and residents to understand the CMP communities’ culture and needs, explore their interest to participate in the development of the research study, and address their concerns were our initial steps in the process of building mutual trust. We also built trust over time by actively listening to community voices, incorporating their voices throughout the research process, and by demonstrating genuine interest in these community stakeholders’ and interviewers’ lives and needs. For example, the research team celebrated the community interviewers’ birthdays, they consistently praised the interviewers’ contributions to the study’s progress, and monitored and responded to the interviewers’ and community stakeholders’ needs whenever is possible. This included connecting community members with disabilities with available AT resources in Puerto Rico, providing transportation support to conduct some of the interviews, or providing supplies to community members affected by Hurricane Maria, and more recently, the COVID19 public health crisis. Likewise, we built trust by taking time to get to know the community members as individuals facilitating the development of personal relationships and rapport. Therefore, we engaged in close physical contact (such as hugging and kissing on the cheek) with the stakeholders and interviewers to greet them, saying good-bye, or acknowledging their outcomes (see Fig. 2). As a result of engaging in these trust building practices, we have been able to sustain mutual trust between the researchers and the community since year 2016.

### Establishing and Maintaining Presence in the Community

The research team was always visible and engaged in community activities to reflect their commitment to making themselves available to the community, even during the long period of time that the funding agency was reviewing the grant application. Therefore, researchers took time to participate in the community’s bingo, annual festival, monthly artisan market, community bike tours, and health fairs. They also assured their continuous presence in the community by co-designing a community-based clinical practice course in occupational therapy for older adults of the CMP communities during the summer terms. Having the research team accessible in these community settings increased the frequency and richness of interactions and contributed to the team’s commitment to listen and be responsive to stakeholders’ concerns.

### Power Sharing

**Fig. 3.**
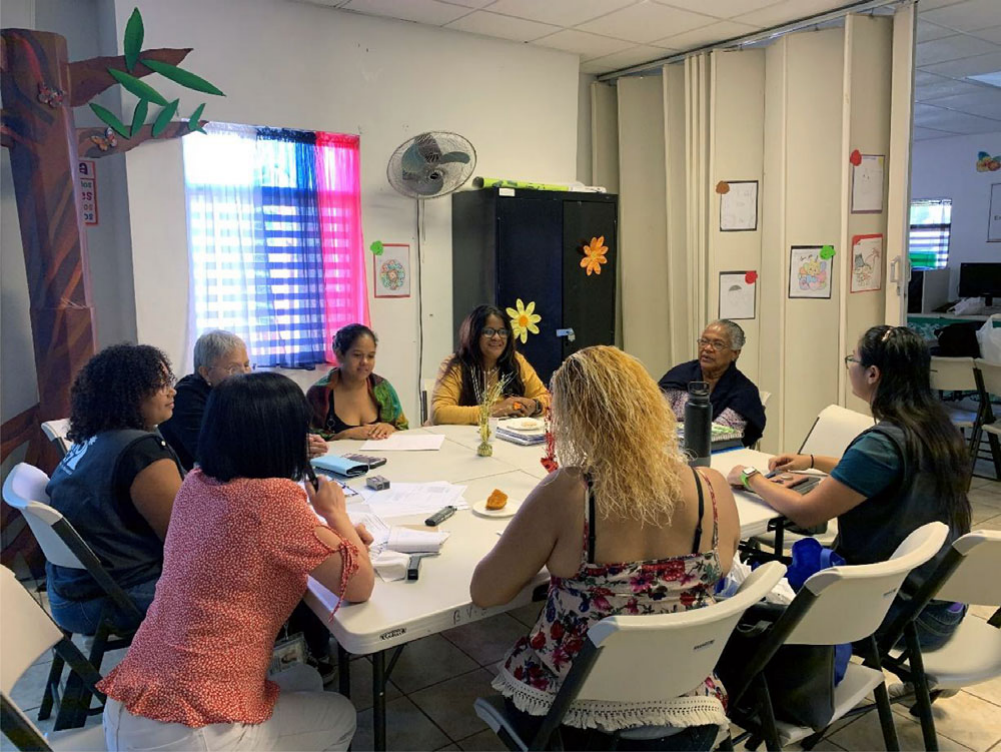
Researcher leaning forward, active listening, and making eye contact with community members during interviewers’ and researchers’ team meetings.

Power sharing is operationalized for the purpose of this study as the researchers’ actions to balance power and share control over the research process. Power sharing was essential for establishing a common ground, resolving study implementation challenges, and supporting meaningful engagement, teamwork, and collaboration. Researchers employed several actions to balance power, such as dispensing with academic titles (by addressing everyone by their first name), dispensing with formal clothing during the working groups, CAB meetings and the recruitment process within the community (by dressing with jeans and sneakers). The researchers also encouraged community stakeholders to speak and contribute their ideas, as well as incorporated their ideas and unique expertise throughout the research process. In each meeting, a shared responsibility for decision-making was always adopted. The researchers used body language to show honest interest in stakeholders’ input, such as nodding, leaning forward, and making eye contact, as well as paraphrasing their contributions to show understanding (see Fig. 3). Finally, we use a circular seating configuration with no head-at-the-table positions during our meetings to demonstrate that the researchers and the community members were equal partners in this study.

### Shared Language

**Fig. 4.**
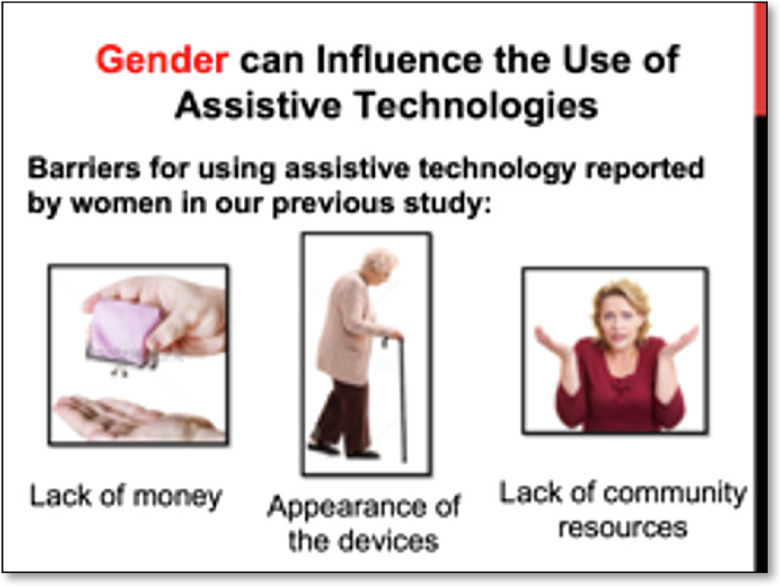
PowerPoint slide with minimum text and visual support for the community interviewers’ training.

Shared language in the context of this study refers to the use of a culturally competent communication that increases the understanding between the researchers and the community stakeholders. For this purpose, the researchers employed clear, plain, simple language, free of technical jargon when communicating with the community stakeholders as well in every study materials for the participants and community members. For example, the stakeholders made changes to improve the clarity of ambiguous statements to community members, such as “persons with disabilities” to a wording more easily understood by the community: “persons with difficulties performing activities of daily living.” They also recommended keeping the sentences as short as possible and adding bullets to long paragraphs. Changes were also necessary to increase the understandability of AT devices listed in the socio-demographic questionnaire. To illustrate this, uncommon references such as “enlarged print documents” was changed to “documents with big letters” and “pill reminders” to “alarms for medications.” Community interviewers input was also instrumental in increasing the cultural competence of the data collection instruments. For example, in the sociodemographic questionnaire, we were able to add relevant examples to items using formal wording and phrases (such as “Payments for informal work”) with familiar language and colloquialisms used by older adults of the CMP communities, such as “chiripas” or “chivitos” to refer to “side jobs.” Finally, lengthy PowerPoint presentations during the stakeholders and CAB members training, as well as during the interviewers’ training that included highly technical language, were also edited to keep them as brief as possible. Visual strategies were used to present complex concepts and to make sure the language and images were clear and easy to understand (see Fig. 4).

### Ongoing Mentorship and Support to Community Members

Ongoing supervision, mentorship, and support were woven throughout the research process using several strategies. The mentorship process was initiated with the comprehensive training provided to the community interviewers to build their capacity to conduct the recruitment and data collection process. Moreover, each community interviewer conducted interviews with another community interviewer as an opportunity to safely practice the entire interview process before going into the field. Once in the field, the interviewers’ first few interviews were conducted in the company of one of the researchers to continue reinforcing the development of interviewing skills and assure data quality. As enrollment progressed, interviewers’ and researchers’ team meetings were held every 2 months, to foster continual learning, support, and problem-solve implementation challenges. As part of these meetings, data quality and recruitment reports were presented and discussed with the community interviewers to troubleshoot study challenges, such as difficulties in reading the community map to identify the selected houses for the study. Regular one-on-one check-ins with an interviewer and a member of the research staff to debrief, discuss concerns, ensure data quality, and address individual training needs were regularly held. The use of the WhatsApp Chat Messaging System was also crucial in providing continuous follow-up to the interviewers, informing about the progress of the enrollment process, providing constant motivation to achieve the weekly goals, clarifying interviewers’ concerns, and acknowledging the interviewers’ achievements. All interviewers recognized the positive impact of the messaging system in supporting their work, as expressed by one of the interviewer: “*One is more motivated because you (the researchers) give us that encouragement and enthusiasm that is important in the kind of work we are doing*.” Refresher videos were also sent as needed through the messaging system to revisit pertinent interviewers’ skills.

### Adapting to the Changing Needs of the Community

Addressing the changing needs of stakeholders and community interviewers facilitated their participation in the research process. For example, to accommodate for childcare challenges, a flexible schedule was implemented to conduct the interviews during the evenings and weekends. Appropriate researchers’ outreach at the interviewers’ convenient hours through telephone calls or the messaging system was also assured to accommodate for the complex life situations of the community interviewers. Transportation challenges were accommodated as well by conducting all the meetings with the community stakeholders and the interviewers’ training within community venues, such as at the conference room of the *G-8* headquarters or at one of the CMP community centers. Finally, research processes that were difficult to implement were also simplified according to the interviewers’ requests. This is to overcome the interviewers’ difficulties in correctly identifying the selected houses for this study, the researchers modified the community maps by sending Google Maps screen shots of the selected study houses to the interviewers, and adding a table with the residential house number besides of the study’s assigned number of the selected houses. Moreover, 3 weeks before the first reported case of coronavirus in Puerto Rico, one of the interviewers expressed her need for information about preventive strategies while conducting the interviews: “Under these circumstances (Coronavirus warnings) how are we going to manage the interviews? That worries me a little, please guide me, because as one has close contact with the participants, I wanted to know.” Our response to this concern was to prepare an anti-coronavirus kit that was distributed to each interviewer with a variety of preventive supplies such as surgical facemasks, hand sanitizers, disinfecting wipes, hand wipes hand gloves, cough drops, alcohol, multivitamins, Tylenols, and alcohol. We also created a picture-based handout with specific prevention practices while conducting the data collection in the community. As expressed by one of the participants in our WhatsApp Chat, we can assert that the interviewer’s concerns were met: “Thank you! Now I feel prepared with all the information that you gave me.”

## Discussion

To address the need for more information about CE in AT research, we described effective practice guidance we used in our AT research study to enhance CE of low-income Hispanic communities in AT research. Our findings highlight the effort, presence, resources, and flexibility required to conduct effective CE. The CE practices employed in this study meet several of the evidence-based indicators for evaluating the contribution of CE to ethical goals in health research that were developed by an international group of experts in behavioral health science, bioethics, and global health [23]. For example, the CAB continues input to enhance the implementation of the study supports the ethical goals of *broadly protect communities in research* and *minimize the possibility of exploitation*. The ethical goal of *ensuring awareness of and respect for cultural differences and for recruited participants* is being met by this study practices of establishing and maintaining presence in the community, building trusting and warm relationships, and using a share language that accommodates the cultural intricacies of the CMP communities. Moreover, the ethical goals concerning a *share responsibility of partners for the conduct of research*, the *minimization of community disruption*, and the *legitimation of the engagement process* are being achieved through two of this study’s CE practice. The first CE practice relates to obtaining early and continuous input from this study stakeholder which was crucial to enhance the study’s protocol, materials, and implementation challenges. The second CE practice concerns to power sharing which provides a mechanism for community members to discuss concerns, share suggestions for research, incorporate the community voices, and resolve conflicts.

This study’s results are also consistent with the findings obtained from 47 funded projects by the Patient-Centered Outcomes Research Institute (PCORI) which identified the value of early and continuous community members’ contributions to the CE process [24]. In our study, early community input allowed the successful implementation and analysis of our pilot preliminary study, as well as the development of a successful funded application. Moreover, continuous community input was instrumental for overcoming implementation challenges during the *Conducting the Research Phase*, as well for enhancing the appropriateness of study materials.

As seen in recent studies, continuous researchers’ efforts in building trusting relationships were an important enabler of CE that supported the success of this study [25–28]. Being trustworthy in Latino (people who are from or descends from people from Latin America) culture implies more than being honest and reliable. Trust in Latino’s is based mostly on personal connections and rapport [29]. It is the perception that a person “knows us” or “is one of us.” We achieved trust by nurturing relationships between the researchers and community members through time and by maintaining ongoing presence in the community, even during times when no research activities were being conducted. Moreover, mutual trust was enhanced by taking actions to balance power such as active listening, considering and incorporating community members’ ideas, and adopting a perspective of shared responsibility for decision making.

While most of the principles that emerged from this study reinforce well-established principles of CE [18,24,26,30], the importance of developing a personal relationship between the researchers and the community members as a fundamental prerequisite for trust was a notable finding. This could be explained by the traditional perception of the patient–health care relationship in the Hispanic and Latino’s culture of “*Personalismo*,” which is a desire for a formal friendliness, good rapport, and personal connection in this relationship [31–33]. While no studies have assessed the translation of “*Personalismo*” to the perception of the community–researcher relationship, such concept may still be pertinent to explain the community’s preference for a personal connection. This points to the need to develop the necessary cultural competence and sensitivity among researchers that target Hispanic and Latino’s populations in their AT research studies, as well as to invest sufficient time to strengthen the partnership with community stakeholders.

Community stakeholders have enormous potential for informing AT research by sharing their expert knowledge and lived experiences, but this is a practice that is often underused by AT researchers. Community stakeholders’ experiential knowledge can improve the quality and relevance of research and enhance research design, implementation, interpretation, and dissemination through the eyes of individuals that represents the study’s population of interest [34]. For example, by engaging in a collaborative community-based approach to conduct the AT study, the researchers were able to support the meaningful involvement of the community interviewers, successfully recruit the study’s participants, and successfully overcome the implementation challenges.

This research has three main limitations. First, we did not conduct quantitative surveys or interviews with community members and stakeholders, thus the opinions reflected in this paper could be skewed toward the researcher perspective. However, two community stakeholders, who are co-authors of this paper, reviewed and provided their recommendations to assure the accuracy of the information presented in the article and to validate the resulting CE principles that emerged from this study. More investigation is needed on identifying effective CE principles and practices from the perspectives of community members and stakeholders. Second, the Hispanic researchers of this study, all the community interviewers, and the majority of the stakeholders were women. There may be different CE practices with male community members based on gender differences and considerations. For example, male researcher could have refrained from engaging in close physical contact with community members and stakeholders due to the traditional *Machismo* ideology, which in part characterize Hispanic males as having reserved emotions [35,36] Engagement of communities in research demands constant reflexivity and an understanding that there may be important different perspectives unique to the characteristics of the community members that must be constantly considered. Third, the CMP communities are a very distinct empowered community in Puerto Rico. Therefore, the practices found to enhance CE within this community may not be generalizable to other low-income Hispanic communities. However, the CE practices found to be effective with the CMP communities may be transferable to other Hispanic and Latino’s communities that shared cultural values such as trustworthiness and *Personalismo* as facilitators of Latino and Hispanic communities in research projects.

This paper describes our experience engaging Hispanic communities in AT research that can help others develop more effective CE in AT studies. It highlights the importance of understanding and validating the communities’ history as a precursor of understanding, contextualizing, and integrating the community’s voices in the research process. This paper also highlights important practices, such as building and trusting personal connections and rapport that can help AT researchers develop more effective CE studies with Hispanic communities. We believe that these practices can enhance the interaction of researchers and community partners to improve the effectiveness of researchers’ efforts to engage marginalized low-income Hispanic communities in their projects. Future research is needed to test and expand these practices with other minority communities as well as with other approaches of CE in AT research.
